# The liver–muscle axis in metabolic dysfunction-associated steatotic liver disease: a narrative review and conceptual framework for body-composition phenotyping

**DOI:** 10.3389/fmed.2026.1954630

**Published:** 2026-09-28

**Authors:** Zhendong Ma, Xinri Zhang, Yan Leng, Xingyu Chen

**Affiliations:** 1 College of Traditional Chinese Medicine, Changchun University of Traditional Chinese Medicine, Changchun, Jilin, China; 2 Department of Liver, Spleen and Gastroenterology, Affiliated Hospital of Changchun University of Traditional Chinese Medicine, Changchun, Jilin, China

**Keywords:** body composition, liver histology, MASH, MASLD, myosteatosis, risk characterization, sarcopenic obesity

## Abstract

Metabolic dysfunction-associated steatotic liver disease (MASLD) exhibits substantial phenotypic heterogeneity, yet pathways centered on body mass index (BMI) and liver non-invasive tests (NITs) do not adequately identify reduced metabolic reserve, functional vulnerability, or the risk of treatment-related muscle loss. This review aims to delineate the conceptual boundaries among sarcopenic obesity (SO), non-obese sarcopenia, and myosteatosis; evaluate their mechanistic, histological, and prognostic relationships with MASLD; compare body-composition assessment tools; and propose a body composition risk characterization framework for future validation. A formal diagnosis of SO should not be based on a mechanical combination of BMI and a single muscle mass threshold, but should concurrently establish excess adiposity, impaired muscle function, and reduced skeletal muscle mass/quantity confirmed by body-composition assessment. Current evidence indicates that low muscle mass, low muscle strength, and myosteatosis are associated with metabolic dysfunction-associated steatohepatitis (MASH), liver fibrosis, and adverse outcomes, although observational associations remain influenced by reverse causation, physical activity, visceral adiposity, and disease stage. Compartment-specific apolipoprotein J (ApoJ/clusterin) signaling illustrates liver-to-muscle endocrine communication and context-dependent intrahepatic or remote-organ effects. We propose four muscle phenotypes: phenotype A, preserved muscle function/quantity without myosteatosis; phenotype B, isolated myosteatosis; phenotype C, abnormal muscle function and/or quantity without myosteatosis; and phenotype D, coexisting abnormalities in muscle function/quantity and myosteatosis. Excess adiposity is treated as an independent modifying axis, and phenotypes C or D are considered SO-related only when excess adiposity is independently confirmed and formal SO criteria are fulfilled, thereby avoiding the misclassification of non-obese sarcopenia as SO. The model is intended for validation in a multicenter prospective biopsy cohort using centrally reviewed, blinded liver histology as the reference for MASH and fibrosis, with its incremental value assessed beyond the fibrosis-4 index (FIB-4), vibration-controlled transient elastography (VCTE), the enhanced liver fibrosis (ELF) test, or magnetic resonance elastography (MRE). Body-composition assessment should complement rather than replace liver NITs, and the four-phenotype classification, selective clinical pathway, and dual liver–muscle endpoint framework remain conceptual, hypothesis-generating tools that require histological, longitudinal-outcome, and clinical-utility validation.

## 1 Introduction

Metabolic dysfunction-associated steatotic liver disease (MASLD) is among the most common chronic liver diseases worldwide, and its burden continues to rise in parallel with obesity, type 2 diabetes (T2D), and population aging (1). The terminology of steatotic liver disease has evolved from nonalcoholic fatty liver disease/nonalcoholic steatohepatitis (NAFLD/NASH), through metabolic dysfunction-associated fatty liver disease (MAFLD), to MASLD/metabolic dysfunction-associated steatohepatitis (MASH) within the current steatotic liver disease (SLD) framework (2–4). MAFLD introduced a positive diagnostic concept centered on metabolic dysfunction and allowed coexisting liver-disease etiologies, whereas MASLD was designed to preserve continuity with the existing NAFLD evidence base while incorporating defined cardiometabolic risk criteria. The current framework also recognizes metabolic dysfunction-associated alcohol-related liver disease (MetALD) when metabolic risk coexists with alcohol exposure above the MASLD threshold (3, 4). Because terminology differs across the literature, this review retains NAFLD, MAFLD, or NASH when describing original studies and uses MASLD/MASH for synthesis and general discussion.

Fibrosis stage remains the major determinant of liver-related outcomes in MASLD, and current clinical pathways therefore rely on liver-directed non-invasive tests (NITs). The fibrosis-4 index (FIB-4) is commonly used as an initial risk-stratification tool, with vibration-controlled transient elastography (VCTE), the enhanced liver fibrosis (ELF) test, or magnetic resonance elastography (MRE) used when further fibrosis assessment is required (5–7). These tools are essential for estimating hepatic fibrosis risk, but they do not directly assess skeletal muscle reserve, physical function, or vulnerability to treatment-related muscle loss. BMI is similarly incomplete because it cannot distinguish visceral from subcutaneous adiposity, lean tissue from fat mass, or metabolically adverse ectopic fat from overall body size. Consequently, patients with normal body weight may still have substantial visceral or intramuscular fat (8–10), whereas patients with similar BMI values may differ markedly in muscle reserve, functional capacity, and prognosis (11–13). These limitations provide the clinical rationale for considering body composition as a complementary, rather than competing, dimension of MASLD risk assessment.

Evidence on the liver–muscle axis has also evolved beyond the simple question of whether low muscle mass accompanies fatty liver. Biopsy studies have linked low muscle mass to MASH and significant fibrosis (14, 15). Quantitative computed tomography (CT) studies have associated low muscle radiodensity or increased muscle fat with histological severity and disease progression (16–19), whereas magnetic resonance imaging (MRI)-based approaches provide complementary characterization of muscle fat (20). Longitudinal cohorts have related low muscle strength or adverse muscle composition to mortality (11, 21). However, muscle mass, muscle strength, and myosteatosis represent distinct biological and clinical constructs and should not be treated as interchangeable markers. In several analyses, associations based on muscle mass weaken after adjustment for obesity, diabetes, or visceral adiposity, whereas selected associations involving muscle composition remain more stable (17–19, 21). This apparent difference may reflect greater biological relevance of muscle composition or function, but it may also arise from indexing methods, measurement error, residual confounding, and study selection. A meaningful synthesis therefore requires comparison of these dimensions rather than simple aggregation of positive associations.

From a clinical perspective, the potential value of body-composition assessment is specific and should not be overstated. It should not replace fibrosis assessment or established liver NITs. Instead, it may address three complementary questions that liver-directed tools do not directly answer: 1) whether the patient has reduced metabolic or functional reserve; 2) whether risk is driven predominantly by excess adiposity, insufficient muscle, abnormal muscle fat, or a combination of these features; and 3) whether potent weight-loss treatment may produce clinically relevant muscle vulnerability. This distinction is particularly important because sarcopenic obesity (SO), non-obese sarcopenia, and myosteatosis overlap but are not equivalent phenotypes. Failure to separate these constructs can obscure both biological interpretation and clinical risk characterization.

Accordingly, this narrative review addresses five scientific questions: 1) how should SO, non-obese sarcopenia, and myosteatosis be distinguished in MASLD; 2) how do adipose tissue, skeletal muscle, and the liver form bidirectional and stage-dependent pathological relationships; 3) what level of evidence—human histology, longitudinal observation, intervention studies, genetic inference, or basic experiments—supports each mechanistic and clinical claim; 4) in which settings are different body-composition tools valid, interpretable, and sufficiently reliable for clinical or research use; and 5) does body-composition information provide incremental value beyond established liver NITs? Clinical application is considered in three predefined settings: prognostic characterization of liver-related and mortality risk, identification of frailty or nutritional vulnerability, and monitoring of muscle safety during potent weight-loss therapy. The principal contribution of this review is a four-category muscle-phenotype framework based on muscle function/quantity and myosteatosis, with excess adiposity treated as an independent modifying axis. We further integrate stage-dependent liver-muscle causality and compartment-specific apolipoprotein J (ApoJ/clusterin) signaling as mechanistic anchors, and propose liver histology as the research reference for future validation. These frameworks are intended to support hypothesis generation, clinical communication, and prospective validation; they are not established diagnostic algorithms and should complement, rather than compete with, current liver risk-stratification systems.

## 2 Literature search and evidence selection

This article is a structured narrative review. PubMed/MEDLINE was searched from database inception through July 27, 2026, using combinations of terms related to “MASLD,” “NAFLD,” “MAFLD,” “MASH,” “NASH,” “sarcopenia,” “sarcopenic obesity,” “myosteatosis,” “muscle attenuation,” “muscle quality,” “body composition,” “visceral adiposity,” “fibrosis,” “mortality,” “exercise,” “nutrition,” “pharmacotherapy,” “bariatric surgery,” “ApoJ,” “clusterin,” “hepatokine,” “low-density lipoprotein receptor-related protein 2 (LRP2),” “mechanistic target of rapamycin (mTOR),” “transcription factor EB (TFEB),” “signal transducer and activator of transcription 3 (STAT3),” “handgrip,” “chair stand,” and “imaging validity/reliability.” Reference lists of major guidelines, consensus statements, systematic reviews, and representative studies were additionally hand-searched, and bibliographic records were verified using digital object identifiers (DOIs), PubMed entries, and journal websites.

Evidence was prioritized in the following order: international guidelines or consensus statements, systematic reviews and meta-analyses, randomized controlled trials, prospective or longitudinal cohorts, liver histology or quantitative-imaging studies, and then cross-sectional and mechanistic studies. The mechanistic section further distinguishes four evidence categories: human longitudinal or histological evidence for assessing temporal sequence and disease severity, cross-sectional studies for describing phenotypic associations, Mendelian randomization and mediation analyses for exploring possible causal directions, and animal or cellular studies solely for demonstrating biological plausibility. Because this was not a preregistered systematic review, dual independent screening, formal risk-of-bias assessment, and quantitative pooling were not performed. The four phenotypes and histology-based validation plan proposed here constitute a conceptual research framework derived from existing evidence rather than a ready-made model already validated in the retrieved literature; for inconsistent findings, emphasis is placed on differences in definitions, muscle mass indexing, imaging parameters, disease stage, visceral fat, T2D, physical activity, and control of other confounders.

## 3 Operational definitions of muscle and adiposity abnormalities

Muscle and adiposity abnormalities in metabolic dysfunction-associated steatotic liver disease (MASLD) cannot be adequately represented by a single label such as sarcopenia, obesity, or “muscle quality.” Muscle function, muscle quantity, myosteatosis, and excess adiposity are related but biologically distinct dimensions, and their definitions and measurement methods vary substantially across studies. This review therefore defines these dimensions separately before integrating them into the four-phenotype framework presented in Section 4.

### 3.1 Muscle function and physical performance

Modern sarcopenia assessment has moved beyond a one-dimensional focus on low muscle mass. The revised European Working Group on Sarcopenia in Older People (EWGSOP2) uses low muscle strength to identify probable sarcopenia, confirms the diagnosis with low muscle quantity or quality, and uses impaired physical performance to indicate greater severity. The Asian Working Group for Sarcopenia (AWGS) 2019 similarly incorporates grip strength, gait speed, and the five-times sit-to-stand test using regionally relevant thresholds (22, 23). These frameworks support a multidimensional approach in which muscle strength, muscle quantity, and physical performance are related but non-equivalent domains.

Muscle strength may be more closely related to patient-important function than muscle quantity alone, but current evidence does not establish universal superiority of one functional measure. Low grip strength has been associated with NAFLD (24), FIB-4-defined advanced fibrosis (25), and mortality (11), while low muscle quantity and low strength may also show independent and additive associations with NAFLD (24). These findings support objective functional testing but do not justify treating a single strength measure as a surrogate for whole-body function. In particular, handgrip primarily reflects upper-limb force and may fail to identify preferential lower-limb impairment (26). The five-times sit-to-stand test, gait speed, or the Short Physical Performance Battery (SPPB) may therefore provide complementary information on lower-limb or global physical performance; this issue is discussed further in Section 7.1.

For clinical and research reporting, muscle function should therefore be identified according to the actual test performed rather than summarized under a nonspecific label. The anatomical region assessed, testing protocol, equipment, threshold source, and relevant limitations should be reported whenever functional abnormalities are used for phenotypic classification.

### 3.2 Muscle quantity and the problem of indexing

MASLD studies use heterogeneous definitions of low muscle quantity. Approaches include appendicular lean mass (ALM) indexed to body mass index (BMI), appendicular skeletal muscle mass indexed to body weight, and sex-specific muscle indices derived from dual-energy x-ray absorptiometry (DXA) or bioelectrical impedance analysis (BIA) (14, 15, 27, 28). Computed tomography (CT)-based skeletal muscle index (SMI) is also used in imaging cohorts (17–19, 29). These choices can materially alter both estimated prevalence and observed associations with liver outcomes. A label such as “low muscle mass” or “sarcopenia” is therefore difficult to interpret unless the measured compartment, denominator, threshold source, and confirmation process are explicitly reported.

Muscle-mass indexing is a particularly important source of heterogeneity. In the Third National Health and Nutrition Examination Survey (NHANES III), height-adjusted and weight-adjusted SMI produced associations with severe steatosis in opposite directions (30), a discrepancy more plausibly explained by denominator choice than by opposing biological effects. Height adjustment primarily reflects muscle reserve relative to stature but may fail to identify muscle that is inadequate for a large body size. In contrast, weight- or BMI-adjustment identifies relative muscle insufficiency but mathematically incorporates adiposity into the exposure itself.

Studies should therefore avoid defining muscle insufficiency from a single denominator. Where feasible, absolute muscle quantity should be reported together with height-adjusted and BMI- or weight-adjusted values. NHANES III directly demonstrated that index choice can materially alter, and even reverse, observed associations between sarcopenia and severe steatosis (30), while prospective, biopsy-based, and sex-stratified studies further show substantial variation across muscle definitions and patient subgroups (31–35).

The European Society for Clinical Nutrition and Metabolism–European Association for the Study of Obesity (ESPEN–EASO) consensus is particularly important when muscle quantity is interpreted in the context of sarcopenic obesity (SO). Its screening-diagnosis-staging framework requires impaired muscle function to be established and body-composition assessment to confirm excess adiposity together with reduced skeletal muscle mass/quantity (36). Mechanically combining an elevated BMI with a single low-muscle-mass threshold is therefore insufficient for a formal diagnosis of SO. Patients with incomplete assessment should be described as being at risk of SO or as having probable SO, whereas confirmed SO should be reserved for those in whom the required functional and body-composition criteria are established.

### 3.3 Myosteatosis: definition, functional relevance, and measurement

Myosteatosis represents a distinct dimension of muscle health. It is an umbrella term describing abnormal lipid accumulation within and around skeletal muscle and may include intermuscular adipose tissue (IMAT), lipid between muscle fascicles or fibers, and intracellular lipid droplets. These compartments are biologically different and should not automatically be treated as interchangeable manifestations of a single process.

Myosteatosis may influence muscle function through several mechanisms (20, 37). Increased lipid infiltration can reduce the relative density of contractile tissue, alter force transmission, impair mitochondrial oxidation and insulin signaling, and create a local adipokine- and cytokine-rich environment that promotes inflammation and anabolic resistance (38–43). This is particularly relevant to MASLD because hepatic and muscular ectopic fat may arise from a shared failure of lipid buffering and may reinforce systemic insulin resistance.

Evidence from chronic liver disease further suggests that the biological meaning of muscle fat may depend on the disease environment. Greater quadriceps IMAT was associated with lower knee-extensor strength and habitual physical activity. Transcriptomic analyses further identified distinct expression profiles in low- and high-IMAT chronic liver disease, with enrichment of atrophic and pro-inflammatory pathways in the high-IMAT group (44). Although these observations are not restricted to MASLD, they suggest that similar amounts of muscle fat may have different functional and inflammatory implications in chronic liver disease.

Measurement method is therefore critical. Reduced mean CT attenuation is an indirect imaging surrogate of muscle composition (19, 45, 46). Dixon magnetic resonance imaging (MRI) estimates regional proton-density fat fraction, whereas magnetic resonance spectroscopy (MRS) can quantify selected local lipid pools (20). These methods capture different biological levels and should not be treated as interchangeable measures of “muscle quality.”

Myosteatosis can occur despite muscle quantity remaining within the reference range and should therefore not be equated with conventional sarcopenia. CT-derived SMI and muscle radiodensity reflect muscle quantity and composition, respectively, and may show different associations with MASLD outcomes (19). Low CT attenuation is additionally influenced by tube voltage, contrast phase, reconstruction technique, anatomical level, muscle selection, age, sex, and BMI (19, 20, 46). It should therefore be interpreted as an imaging phenotype rather than as a histological diagnosis.

No globally transportable CT attenuation threshold currently exists because attenuation varies with protocol, anatomical level, sex, age, and adiposity (16, 19, 20, 46). MRI-derived muscle fat fraction more directly estimates tissue lipid but remains dependent on sequence, field strength, region-of-interest selection, and analysis software (20). Reference values should therefore be protocol- and population-specific, and categorical thresholds should be accompanied by continuous measurements.

For the same reason, the term “muscle quality” should not be used as a synonym for strength, CT attenuation, MRI fat fraction, strength-to-mass ratios, or BIA phase angle because these measures represent different biological constructs (47). Throughout this review, the actual measured variable is therefore preferred over the nonspecific term “muscle quality.”

### 3.4 Excess adiposity and visceral Fat distribution

Adiposity represents another independent dimension of the MASLD phenotype. BMI is practical for population-level screening but cannot distinguish fat mass from lean tissue or identify fat distribution. Waist circumference better reflects central obesity but varies with height, sex, and ethnicity. Total body fat percentage estimates overall adiposity but does not directly distinguish visceral from subcutaneous fat. CT- or MRI-derived visceral fat provides more direct anatomical information but is less suitable for universal screening because of cost, accessibility, and, for CT, radiation exposure.

These differences are clinically important because metabolic risk is not determined by BMI alone. Measures combining muscle and adiposity, including muscle-to-visceral-fat and skeletal-muscle-to-body-fat ratios, have been associated with liver fibrosis, cardiometabolic abnormalities, and mortality in MASLD or related NAFLD populations (12, 48–54). Such observations support a layered description of total adiposity, visceral fat distribution, muscle reserve, and muscle function rather than classification based exclusively on BMI or obesity class.

Normal body weight likewise does not imply low metabolic risk. Lean or non-obese NAFLD can coexist with increased visceral adiposity, sarcopenia, and myosteatosis, and these abnormalities are associated with adverse metabolic and long-term outcomes (9, 10). Patients with normal BMI but increased waist circumference, type 2 diabetes (T2D), reduced muscle strength, or imaging-defined myosteatosis may therefore remain metabolically high risk. Conversely, individuals with obesity but preserved muscle reserve and function may differ substantially from patients in whom excess adiposity coexists with muscle insufficiency.

For the phenotype framework developed in this review, excess adiposity is therefore treated as an independent modifying dimension rather than as part of the primary A–D muscle classification. This separation is essential because muscle insufficiency can occur without obesity, obesity can occur with preserved muscle function and quantity, and myosteatosis can occur with or without generalized excess adiposity.

No MASLD-specific diagnostic threshold or staging system for SO currently exists. Studies vary in the use of BMI, waist circumference, body fat percentage, or visceral fat to define adiposity and in the use of height-, weight-, or BMI-indexed measures to define low muscle quantity (30, 36, 55). A patient should therefore first be characterized according to the muscle dimensions actually measured; the presence or absence of excess adiposity should then be established independently. This two-step principle provides the basis for the four-phenotype framework described in Section 4.

## 4 A four-phenotype framework for muscle abnormalities in MASLD

### 4.1 Rationale for separating muscle phenotype from adiposity status

The proposed framework separates muscle phenotype from adiposity status because SO, non-obese sarcopenia, muscle insufficiency, and myosteatosis are overlapping but non-equivalent conditions. Phenotypes A–D are defined only by (i) abnormal muscle function and/or quantity and (ii) myosteatosis; excess adiposity is assessed separately.

Assignment to phenotype C or D therefore does not establish SO. A non-obese patient with reduced muscle function or quantity remains non-obese sarcopenia or muscle insufficiency, whereas excess adiposity with isolated myosteatosis (phenotype B) is not SO unless the required muscle criteria are also fulfilled. Formal SO requires independently confirmed excess adiposity, impaired muscle function, and reduced skeletal muscle mass/quantity according to accepted diagnostic criteria (36).

### 4.2 Definition of the four muscle phenotypes

1. Phenotype A: preserved muscle function/quantity without myosteatosis

Phenotype A is defined by preserved muscle function and quantity without evidence of myosteatosis using the available assessment method. It represents the reference muscle phenotype within the proposed matrix.

Phenotype A does not imply absence of obesity or liver disease; adiposity status and liver stage should therefore be recorded independently.

2. Phenotype B: isolated myosteatosis

Phenotype B is characterized by myosteatosis in the presence of otherwise preserved muscle function and quantity. This phenotype is conceptually important because compositional abnormalities may occur before overt loss of muscle quantity or measurable functional impairment.

Preclinical studies indicate that myosteatosis can occur early and may precede overt muscle atrophy (37, 56), whereas a prospective human biopsy cohort associated severe myosteatosis with early NASH and subsequent fibrosis progression (18). Phenotype B may therefore represent an early or composition-dominant muscle abnormality, although this interpretation remains hypothesis-generating and requires prospective validation.

Phenotype B should not be classified as sarcopenia on the basis of muscle fat alone, and excess adiposity plus phenotype B should not be classified as SO without the required functional and muscle-quantity criteria.

3. Phenotype C: muscle insufficiency without myosteatosis

Phenotype C is defined by abnormal muscle function and/or quantity without measured myosteatosis. It identifies patients in whom reduced functional or quantitative muscle reserve is the dominant abnormality.

Interpretation of phenotype C depends on adiposity status. Without excess adiposity it represents non-obese sarcopenia or muscle insufficiency, according to the criteria fulfilled; with independently confirmed excess adiposity it may be considered SO-related only when formal SO criteria are met.

4. Phenotype D: combined muscle insufficiency and myosteatosis

Phenotype D combines abnormal muscle function and/or quantity with myosteatosis. It therefore represents simultaneous impairment of muscle reserve and muscle composition.

Phenotype D may plausibly identify greater combined vulnerability than phenotypes with only one muscle abnormality, but this risk hierarchy remains hypothetical because no prospective cohort has validated all A–D dimensions concurrently (9, 21, 57, 58).

Phenotype D is not synonymous with SO. Without excess adiposity it represents non-obese sarcopenia or muscle insufficiency with myosteatosis; only with independently confirmed excess adiposity and fulfillment of SO criteria should it be considered SO-related.

### 4.3 Excess adiposity as an independent modifier of the four phenotypes

Excess adiposity functions as a third, independent dimension. Classification therefore proceeds in two stages: first assign A–D from muscle function/quantity and myosteatosis; then assess adiposity separately. Table 1 distinguishes sarcopenic obesity, non-obese sarcopenia/muscle insufficiency, and myosteatosis within this adiposity-modified framework, while Figure 1 illustrates the phenotype-by-adiposity interpretation and the two-step classification.

**Figure 1:**
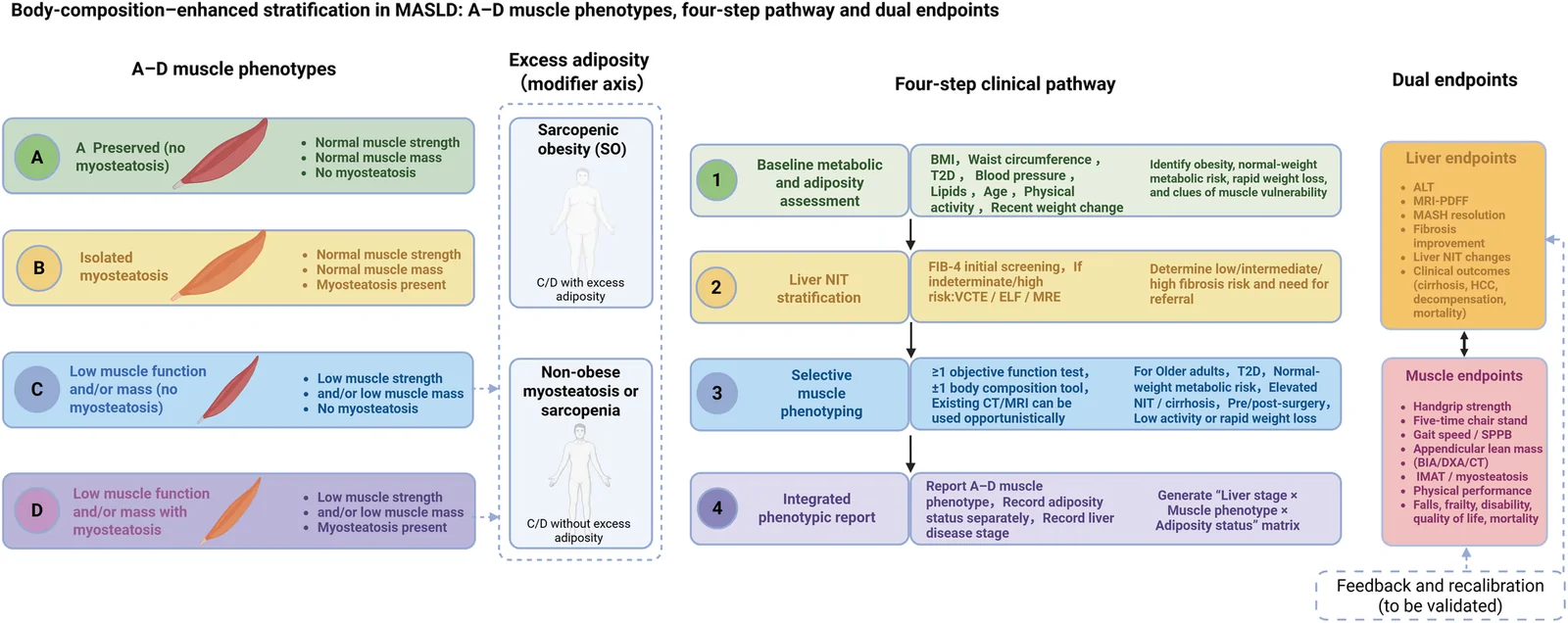
Body-composition-enhanced metabolic dysfunction-associated steatotic liver disease (MASLD) stratification. The four-phenotype framework is defined by muscle function/quantity and myosteatosis: phenotype A, preserved function/quantity without myosteatosis; phenotype B, isolated myosteatosis; phenotype C, abnormal function and/or quantity without myosteatosis; and phenotype D, combined muscle insufficiency and myosteatosis. Excess adiposity is assessed separately: C or D is sarcopenic obesity (SO)-related only when excess adiposity and formal SO criteria are confirmed; otherwise it represents non-obese sarcopenia or muscle insufficiency. The four-step pathway combines metabolic/adiposity assessment, liver non-invasive test (NIT) stratification, selective muscle assessment, and integrated reporting of liver stage × muscle phenotype × adiposity status (5–7, 22, 23, 25, 36, 110, 112–116, 130, 147). The framework requires histological, longitudinal, and clinical-utility validation. Created in BioRender. Ma, Z. (2026). https://BioRender.com/njfpody

**Table 1:** Distinguishing sarcopenic obesity, non-obese sarcopenia/muscle insufficiency, and myosteatosis within the proposed adiposity-modified phenotype framework.

Dimension	Sarcopenic obesity (SO)	Myosteatosis	Meaning in the proposed framework	Adiposity-dependent interpretation
Core concept	Coexistence of excess adiposity and sarcopenia; adiposity and muscle criteria must be established independently.	Ectopic lipid accumulation within or between skeletal muscles; CT attenuation, Dixon MRI fat fraction, and MRS lipid pools reflect different technical levels.	SO and myosteatosis may overlap or occur independently; low CT attenuation is an imaging surrogate, not a histological synonym.	Assign A–D from muscle status first; assess excess adiposity separately as a modifier.
Diagnostic elements	At minimum: excess adiposity, objective impairment of muscle function, and reduced skeletal muscle mass/quantity confirmed by body-composition assessment.	Defined by imaging or spectroscopy; concurrent low muscle mass or strength is not required.	A–D encode muscle status only; SO status is determined after separate adiposity assessment.	A/B with excess adiposity are not SO on adiposity or myosteatosis alone. C/D without excess adiposity remain non-obese sarcopenia/muscle insufficiency; C/D with excess adiposity are SO-related only if formal SO criteria are fulfilled.
Recommended measurements	Objective function (e.g., grip strength or five-times sit-to-stand) + DXA/BIA/CT muscle quantity + BMI, waist circumference, body-fat percentage, or visceral fat.	CT muscle attenuation, Dixon MRI muscle fat fraction, or MRS; ultrasonography may be an adjunct.	Report function, muscle quantity, muscle fat, and adiposity as separate variables.	Assess adiposity separately using BMI, waist circumference, body-fat percentage, or visceral fat.
Reported associations and potential clinical relevance	May identify frailty, falls, nutritional vulnerability, and muscle vulnerability during weight loss.	Reported associations include MASH/fibrosis severity, disease progression, impaired exercise tolerance, and mortality; causal and incremental prognostic value remain uncertain.	Phenotype D may plausibly indicate combined vulnerability, but the A–D risk hierarchy has not been prospectively validated.	Helps distinguish non-obese muscle insufficiency/myosteatosis from SO-related phenotypes without assigning all muscle abnormalities to SO.
Principal uncertainties	Population thresholds for excess adiposity and low muscle mass and methods of muscle-mass indexing are inconsistent.	Imaging level, contrast phase, sequence, software, and thresholds are heterogeneous.	Prioritize continuous values, technical parameters, threshold sources, and prespecified sensitivity analyses.	No MASLD-specific SO threshold exists; adiposity definitions and phenotype-by-adiposity interactions require prospective validation.

SO, sarcopenic obesity. The SO definition is based on sarcopenia and SO consensus statements (22, 23, 36), and technical definitions of myosteatosis are provided in references (19, 20, 45, 46). Phenotypes A–D are defined independently of adiposity. C/D phenotypes are SO-related only when excess adiposity is independently confirmed and formal SO criteria are fulfilled; otherwise they remain non-obese sarcopenia or muscle insufficiency. A/B phenotypes with excess adiposity are not SO on the basis of adiposity or myosteatosis alone. This framework is conceptual and does not constitute a MASLD-specific diagnostic standard.

### 4.4 Classification safeguards and clinical interpretation

Three safeguards are essential. If muscle fat has not been assessed, myosteatosis should be recorded as unknown rather than absent; an isolated abnormal strength result without adequate quantity or composition assessment represents probable functional impairment rather than a fully characterized C/D phenotype; and “SO-related” should not be applied unless excess adiposity is independently confirmed.

Phenotype assignment should describe only dimensions that have actually been measured. Because few cohorts combine objective function, muscle quantity, muscle fat, adiposity, liver histology, and longitudinal outcomes, A–D should be regarded as a research-stratification and clinical-communication framework rather than a validated diagnostic algorithm.

The phenotypes may also be dynamic. Prospective cohorts indicate that NAFLD/MASLD can precede incident sarcopenia or accelerated muscle loss (59, 60), whereas improvement in muscle mass or muscle-to-fat balance has been associated with improvement in liver-related indices (54, 61–63); transitions among A–D should therefore be tested prospectively.

Clinically, assessment should answer four separate questions: 1) Is muscle function impaired? 2) Is muscle quantity reduced? 3) Is myosteatosis present? and 4) Is excess adiposity independently present? Table 1 summarizes the conceptual and measurement distinctions, and Figure 1 illustrates the two-step classification and key safeguards.

## 5 Evidence linking individual muscle dimensions to liver and clinical outcomes

Although Section 4 proposes an integrated A–D framework, most outcome studies evaluate individual muscle dimensions rather than all defining axes concurrently. The evidence below is therefore organized by the muscle abnormality actually measured and the outcome assessed; A–D should not be interpreted as a validated prognostic system.

### 5.1 Liver histology and liver-related outcomes

Available evidence consistently links several dimensions of muscle abnormality to more severe liver disease, but the strength and independence of these associations vary according to the muscle measure, liver-disease stage, and adjustment strategy. At the steatosis stage, low muscle mass, reduced muscle strength, and low muscle radiodensity have each been associated with NAFLD (19, 24, 27). Biopsy-based studies further associate sarcopenia with NASH/MASH and significant or severe fibrosis (14, 15). CT-based cohorts link muscle quantity and composition to significant fibrosis (29), low-attenuation muscle to fibrosis progression (17), and severe myosteatosis to early steatohepatitis and subsequent fibrosis progression (18).

These observations support an association between adverse muscle status and histological severity, but they do not establish a simple causal sequence. Longitudinal studies show that NAFLD can precede subsequent sarcopenia and accelerated muscle loss (59, 60), indicating that liver disease may contribute to deterioration in muscle as well as coexist with pre-existing muscle abnormalities. The relationship is therefore more appropriately interpreted as potentially bidirectional, particularly in non-cirrhotic metabolic disease, rather than as evidence that muscle loss alone drives progression from steatosis to MASH or fibrosis.

The relative importance of muscle quantity, function, and myosteatosis remains uncertain. Associations based on low muscle mass often attenuate after adjustment for BMI, T2D, or visceral adiposity, whereas selected associations involving muscle radiodensity or fat infiltration remain more stable (17–19). This may reflect closer links between myosteatosis, ectopic lipotoxicity, insulin resistance, and local inflammation, but stronger association does not establish biological superiority: indexing, measurement error, imaging protocol, cohort selection, and residual confounding can all alter effect size. Direct head-to-head comparisons within the same population are therefore required.

Interpretation becomes particularly difficult in advanced fibrosis and cirrhosis. Myosteatosis is associated with mortality and other adverse outcomes in cirrhosis, and liver-transplant cohorts associate preoperative myosteatosis with major postoperative complications, prolonged intensive-care or hospital stay, and mortality (64–67). Some direct-comparison studies suggest stronger prognostic performance for myosteatosis than for low muscle mass, but this finding cannot yet be generalized across disease stages or imaging protocols.

Advanced liver disease also introduces substantial reverse causation and measurement complexity. Ascites and edema can distort estimates of muscle quantity, while hyperammonemia, systemic inflammation, inadequate energy or protein intake, reduced physical activity, and repeated hospitalization can directly accelerate muscle loss. Muscle abnormalities in cirrhosis may therefore function simultaneously as markers of disease severity, mediators of functional vulnerability, and consequences of liver failure. Associations derived from advanced cirrhosis should not automatically be extrapolated to early or non-cirrhotic MASLD.

Taken together, the liver-outcome literature supports a clinically relevant relationship between adverse muscle status and MASH, fibrosis, and disease progression, but it does not yet establish one muscle dimension as universally superior or independently causal. Comparisons across studies should therefore distinguish the muscle variable measured, the liver stage studied, and the extent to which adiposity, metabolic disease, physical activity, and reverse causation were addressed.

### 5.2 Mortality and extrahepatic outcomes

The clinical relevance of muscle abnormalities in MASLD extends beyond liver histology. Mortality data provide some of the strongest patient-important evidence, although definitions of both liver disease and muscle abnormality vary substantially across cohorts. Low muscle strength has been associated with increased mortality risk, whereas BMI alone has shown weaker prognostic discrimination in some populations (11). A nationwide Korean survey found higher mortality among individuals with coexisting NAFLD and sarcopenia, and long-term U.S. follow-up similarly associated NAFLD with sarcopenia with increased all-cause mortality (58, 68). Adverse muscle composition, including increased muscle fat, has also been associated with mortality in longitudinal imaging cohorts (21).

These findings suggest that body size or muscle quantity alone may miss functional or compositional vulnerability. However, low strength, low muscle quantity, and myosteatosis should not be assumed to carry equivalent prognostic information because cohorts differ in sarcopenia definitions, indexing, adjustment, imaging selection, and disease stage.

Cardiovascular disease is a particularly important extrahepatic outcome in MASLD. Low muscle mass combined with abdominal obesity has been associated with greater long-term cardiovascular risk (69), while physical inactivity is simultaneously associated with fibrosis, sarcopenia, and cardiovascular risk (70). Among patients with T2D, coexisting NAFLD and sarcopenia has also been associated with progression of carotid atherosclerotic plaque (71). These findings are compatible with a shared cardiometabolic pathway linking visceral adiposity, insulin resistance, impaired muscle reserve, and liver disease.

Muscle abnormalities may also identify vulnerability beyond cardiometabolic disease. MASLD and low muscle strength have been associated with additive fracture risk (72), supporting a broader relationship among liver disease, skeletal fragility, impaired physical function, and frailty. Nevertheless, these associations do not establish that muscle abnormalities are direct causal mediators. Reduced physical activity, aging, nutritional status, diabetes, cardiovascular disease, and liver-disease severity may act as common determinants of both muscle impairment and extrahepatic outcomes.

The extrahepatic evidence base is clinically suggestive but fragmented. Mortality studies often define NAFLD noninvasively and assess muscle with BIA or DXA, whereas myosteatosis studies frequently use smaller imaging-selected populations with more advanced disease. Few cohorts measure strength, muscle quantity, muscle fat, visceral adiposity, histology, activity, and comorbidity concurrently; incremental prognostic value beyond cardiovascular risk factors and liver NITs therefore remains uncertain.

Overall, muscle abnormalities mark multisystem vulnerability, but whether they are causal contributors, mediators, or integrated markers of a shared adverse phenotype remains unresolved.

### 5.3 Effect sizes, confounding, and certainty of evidence

Reported effect sizes suggest clinically meaningful associations between muscle abnormalities and hepatic or extrahepatic outcomes, but their magnitude varies substantially with phenotype definition and confounder control. In biopsy cohorts, adjusted odds ratios linking sarcopenia with NASH and significant or severe fibrosis are approximately 2.0–2.4 (14, 15), while severe myosteatosis has been associated with more than a twofold risk of early NASH and fibrosis progression (18). Longitudinal studies also report persistent associations of low grip strength or increased muscle fat with mortality (11, 21). These findings support clinical relevance but do not establish causality.

Interpretation is complicated because obesity, visceral adiposity, T2D, physical activity, cardiovascular comorbidity, nutritional status, and liver-disease severity may act as confounders, mediators, or both. Adjustment can therefore either reduce bias or remove part of the biological pathway of interest. This problem is especially important for visceral adiposity and physical inactivity, which may contribute to both muscle impairment and liver progression while also changing as disease severity increases.

Representative studies illustrate this sensitivity to model specification. In Koo et al., the association between sarcopenia and overall NAFLD prevalence was attenuated after adjustment for BMI, diabetes, and hypertension, whereas associations with histological NASH and significant fibrosis persisted (14). In Linge et al., the mortality hazard ratio for a composite adverse muscle phenotype decreased from 2.84 to 1.72 after inclusion of physical activity, grip strength, and cardiometabolic comorbidities, while muscle fat infiltration retained an independent association (21). By contrast, additional adjustment for visceral adiposity produced little change in the association between myosteatosis and early NASH in Hsieh et al., and myosteatosis remained associated with fibrosis progression in sensitivity analyses (18). These contrasts suggest that selected functional or compositional measures may be more robust than some muscle-mass indices, but residual confounding, selection bias, and measurement differences remain important.

The certainty of evidence therefore differs across claims. Associations between adverse muscle status and more severe MASLD, mortality, or functional vulnerability are relatively consistent; evidence is weaker that one muscle dimension is consistently superior or independently causal, and weakest for prognostic ranking of A–D and incremental clinical value beyond established liver NITs. Statistical significance alone should not be interpreted as clinical utility; prospective head-to-head studies should use standardized measurement, explicit confounder handling, calibration, decision curves, and net benefit. Representative effect estimates, confounder adjustment, and key evidence limitations are summarized in Table 2.

**Table 2:** Effect estimates, confounder control, and evidence boundaries in representative studies.

Study	Design, exposure, and outcome	Principal effect estimate	Adjustment factors and evidence boundaries
Koo et al., 2017 (14)	Biopsy cohort, *n* = 309; sarcopenia with NASH and significant fibrosis.	NASH: aOR 2.28 (95% CI 1.21–4.30); significant fibrosis: aOR 2.05 (1.01–4.16).	Overall NAFLD association attenuated after adjustment for BMI, diabetes, and hypertension, whereas histological associations persisted; ASM/body weight involves denominator coupling; CT visceral fat and physical activity were not directly adjusted for.
Petta et al., 2017 (15)	Western biopsy cohort, *n* = 225; sarcopenia with severe fibrosis.	Severe fibrosis: 48.3% vs. 20.4%; aOR 2.36 (1.16–4.77).	Association persisted after adjustment for age and glucose-metabolism abnormalities/NASH; visceral fat and physical activity were not measured; BIA, hydration status, and tertiary-center case mix limit generalizability.
Hsieh et al., 2023 (18)	Prospective biopsy cohort, *n* = 338; severe myosteatosis with early NASH and fibrosis progression.	Early NASH: aOR 2.44 (1.22–4.89); fibrosis progression: HR 2.09–2.49.	Adding visceral fat changed the OR very little; progression was defined by repeated VCTE rather than repeat biopsy; physical activity was not measured directly.
Lee et al., 2023 (17)	Longitudinal biopsy-proven NAFLD cohort, *n* = 292; low-quality muscle index with fibrosis progression.	Per quartile increase: HR 1.41 (1.04–1.91); highest vs. lowest quartile: HR 3.25 (1.18–8.90).	Dose-response relationship, but limited events, a single-center CT threshold, unmeasured physical activity, and a VCTE surrogate endpoint increase uncertainty.
Chun et al., 2023 (57)	Development, validation, and longitudinal cohorts, *n* = 23,889; high-risk SO with fibrosis, CVD, and a composite outcome.	Significant fibrosis: aOR 2.38–3.72; CVD: aOR 3.71–5.20; composite outcome: sHR 5.37.	High-risk SO was defined with FIB-4/NFS or ASCVD probability, creating possible structural overlap between exposure and outcome; direct visceral-fat and objective-activity measures were unavailable.
Charatcharoenwitthaya et al., 2022 (11)	Nationwide cohort, *n* = 7,083; low grip strength with mortality.	Men: aHR 3.21 (1.35–7.62); women: aHR 2.22 (1.25–3.93).	Association persisted after adjustment for BMI, activity, and comorbidities; NAFLD was defined noninvasively, activity was questionnaire-based, and reverse causation from functional decline cannot be excluded.
Linge et al., 2023 (21)	UK Biobank MRI cohort; NAFLD *n* = 5,069; muscle composition with mortality.	Composite adverse muscle composition: crude HR 2.84 (1.70–4.75); fully adjusted myosteatosis aHR 1.13 (1.01–1.26).	After adding grip strength, physical activity, and cardiometabolic comorbidities, the composite HR fell to 1.72 (*P* = 0.051); healthy-volunteer bias and relatively short follow-up limit interpretation.
Kim et al., 2024 (45)	Cross-sectional CT study, *n* = 18,154; MASLD with sarcopenia/myosteatosis.	Sarcopenia: aOR 2.62 (1.94–3.54); myosteatosis: aOR 1.75 (1.52–2.02).	Large sample supports phenotypic associations, but the cross-sectional design cannot establish temporal sequence or causal direction.
Kim et al., 2023 (9)	Non-obese population, *n* = 14,400; visceral obesity, sarcopenia, and myosteatosis with NAFLD.	Visceral obesity: OR 3.97–5.42; sarcopenia: OR 1.41–1.59; myosteatosis: OR 1.23–1.24.	Normal BMI does not exclude body composition risk; the outcome was prevalent NAFLD rather than disease progression or clinical events.

aHR, adjusted hazard ratio; aOR, adjusted odds ratio; ASCVD, atherosclerotic cardiovascular disease; ASM, appendicular skeletal muscle mass; CI, confidence interval; CVD, cardiovascular disease; HR, hazard ratio; NFS, NAFLD fibrosis score; sHR, subdistribution hazard ratio. Effect estimates are presented to illustrate the magnitude and limitations of representative associations.

## 6 Biological basis of the bidirectional liver–muscle relationship

### 6.1 Insulin resistance, substrate redistribution, and ectopic lipotoxicity

Skeletal muscle is the principal organ responsible for postprandial glucose disposal and is therefore a major determinant of systemic metabolic homeostasis (38, 41). Reduced muscle quantity decreases total glucose-disposal capacity, whereas myosteatosis may impair insulin sensitivity per unit of muscle tissue; both can increase circulating glucose and insulin and promote hepatic *de novo* lipogenesis (38, 41).

Conversely, hepatic steatosis and adipose-tissue dysfunction increase free-fatty-acid flux and systemic insulin resistance. When adipose storage capacity is exceeded, lipids accumulate ectopically in liver and skeletal muscle; diacylglycerol and ceramides can impair muscle insulin signaling and promote oxidative stress, while analogous lipotoxic pathways in hepatocytes contribute to endoplasmic-reticulum stress, inflammation, and fibrosis (37, 38, 41–43).

Liver fat and muscle fat should therefore be viewed as parallel manifestations of impaired lipid buffering rather than isolated depots. Adipose dysfunction, hepatic fat, muscle fat, and insulin resistance can form a mutually reinforcing metabolic loop (38, 41, 43).

### 6.2 Hepatokines and interorgan endocrine crosstalk: ApoJ as a molecular anchor

Beyond metabolic substrates, the liver communicates with skeletal muscle and distant organs through hepatokines. Among these factors, apolipoprotein J (ApoJ/clusterin) provides a useful molecular example of how hepatic output can exert context-dependent effects across organs.

#### 6.2.1 Physiological liver-derived ApoJ regulates skeletal muscle metabolism

The liver is an important source of circulating ApoJ. Experimental evidence indicates that secreted hepatic ApoJ reaches skeletal muscle, interacts with low-density lipoprotein receptor-related protein 2 (LRP2), facilitates insulin-receptor internalization, and supports downstream insulin receptor substrate/protein kinase B (Akt) signaling and glucose uptake. Loss of hepatic ApoJ or muscle-specific LRP2 disrupts glucose homeostasis and promotes insulin resistance (73).

These findings establish a liver-to-muscle endocrine circuit in which physiological secreted ApoJ contributes to metabolic fitness. Importantly, ApoJ should not be interpreted as a uniformly harmful hepatokine; its biological effect depends on cellular compartment, molecular form, target organ, and disease context.

#### 6.2.2 Stress-induced intracellular ApoJ promotes hepatic lipid accumulation and fibrogenic remodeling

In contrast to physiological secreted ApoJ, stress-induced intracellular ApoJ may contribute to pathological hepatic remodeling. Intracellular ApoJ interacts with mechanistic target of rapamycin (mTOR) and interferes with F-box and WD repeat domain-containing 7 (FBW7)-mediated ubiquitination, stabilizing mTOR and impairing lipophagy. Experimental antagonism of ApoJ chaperone activity promotes proteasomal mTOR degradation and reduces hepatic lipid accumulation (74).

ApoJ also participates in fibrogenic signaling. In injury-induced fibrosis models, hepatocyte ApoJ promotes RAN-binding protein 2 (RANBP2)-mediated SUMOylation of signal transducer and activator of transcription 3 (STAT3), enhancing STAT3 signaling, transforming growth factor-beta signaling, reactive oxygen species production, and hepatic stellate-cell activation (75). These data provide mechanistic plausibility for a role in fibrogenic remodeling but do not establish clinical causality in MASLD.

However, these mechanisms remain predominantly experimental. Their clinical relevance in MASLD requires validation in human longitudinal studies and should not be interpreted as evidence that ApoJ inhibition is universally beneficial.

#### 6.2.3 Liver-to-kidney ApoJ signaling and systemic complications

The impact of hepatic ApoJ extends beyond the liver–muscle axis. Under diabetic metabolic stress, liver-derived ApoJ can accumulate in renal tissue and disrupt transcription factor EB (TFEB)-mediated lipid homeostasis and autophagy, aggravating renal injury (76). This supports ApoJ as an example of liver-derived signaling with remote-organ consequences.

Together, these findings broaden the concept of hepatic-derived metabolic communication. The liver may influence skeletal muscle metabolic fitness and remote-organ vulnerability through circulating factors, with ApoJ illustrating how the same molecule may exert protective or pathological effects depending on biological context.

### 6.3 Inflammatory and endocrine signaling along the liver–adipose–muscle axis

ApoJ represents one hepatokine pathway within a broader inflammatory network. Visceral adipose inflammation can increase tumor necrosis factor alpha (TNF-α), interleukin-6 (IL-6), and related chemokines, while hepatic lipotoxicity activates Kupffer-cell and inflammasome pathways; muscle fat infiltration can add local inflammatory and proteolytic signaling (38, 39, 77). These processes may reinforce insulin resistance, anabolic resistance, and fibrogenesis. Associations of dietary inflammatory burden or circulating leukocyte cell-derived chemotaxin 2 with muscle decline provide additional, but nonspecific, support (78, 79).

The endocrine network is similarly context dependent. Adiponectin and leptin influence insulin sensitivity and inflammatory signaling (38, 55, 80). Myokines such as irisin and myostatin affect mitochondrial adaptation and muscle synthesis (39, 43, 81–83), while hepatokines including fibroblast growth factor 21 (FGF21), fetuin-A, and selenoprotein *P* may influence skeletal muscle and adipose metabolism (55, 81–83). These signals should be interpreted by tissue source, exposure duration, receptor sensitivity, and disease stage rather than as uniformly beneficial or harmful.

Circulating concentrations may be compensatory rather than causal—for example, elevated FGF21 may reflect metabolic stress, while IL-6 has context-dependent effects. Few human studies repeatedly measure upstream exposure, candidate mediators, liver histology, and muscle outcomes in the temporal sequence required for formal mediation analysis. Inflammation is therefore an important network component, but no single factor is established as the dominant causal link.

### 6.4 Mitochondrial dysfunction, oxidative stress, and anabolic resistance

Mitochondrial dysfunction is another shared node between liver and skeletal muscle. Reduced oxidative phosphorylation and incomplete fatty-acid oxidation can generate reactive oxygen species and lipotoxic intermediates; in muscle this reduces oxidative capacity and exercise tolerance, whereas in liver it increases oxidative stress and susceptibility to lipotoxic injury (56, 84, 85). Mechanistic and clinical reviews likewise identify mitochondrial dysfunction, altered myokine signaling, and anabolic resistance as recurrent features of muscle abnormalities in MASLD (41, 43, 81–83).

Experimental studies support biological plausibility. Activation of the retinoic acid receptor-related orphan receptor alpha (ROR*α*)–GA-binding protein (GABP)–mitochondrial transcription factor A (TFAM) axis can reduce myosteatosis by improving mitochondrial capacity (56). Disruption of kallistatin–adiponectin receptor 1 (AdipoR1)–AMP-activated protein kinase (AMPK) signaling promotes myosteatosis and exercise intolerance (86), while adenosine deaminase acting on RNA 2 (ADAR2)–serum amyloid A1 (SAA1) signaling links inflammatory and atrophy programs (87). Human pharmacological validation remains limited.

Anabolic resistance further links metabolic disease to muscle loss. Aging, insulin resistance, chronic inflammation, inadequate protein intake, and cirrhosis-related hyperammonemia can blunt postprandial muscle-protein synthesis, while rapid weight loss may accelerate lean-tissue loss without resistance exercise and adequate protein (88–91). This is clinically relevant when potent weight-loss therapies are used in patients with low baseline muscle reserve.

Mitochondrial dysfunction and anabolic resistance therefore interact, with their relative contribution varying by age, nutrition, adiposity, treatment exposure, and liver stage.

### 6.5 Stage-dependent directionality, reverse causation, and clinical effect modifiers

The direction of the liver–muscle relationship is modified by age, sex, ethnicity, behavior, and liver-disease stage. Aging and sex-hormone changes reduce anabolic and mitochondrial reserve and may increase susceptibility to muscle insufficiency and SO (28, 55, 92). Sex- and region-specific associations further argue against universal body-composition thresholds (12, 32, 33, 93, 94).

Physical activity is another major modifier. Inactivity is associated with sarcopenia, fibrosis, and mortality (70, 95), but may act as both confounder and mediator: it can contribute to metabolic dysfunction early and become a consequence of fatigue, hospitalization, or decompensation later. Repeated or device-based activity measures may therefore be preferable to a single baseline questionnaire when causal relationships are examined (96–98).

For this reason, clinical directionality should be considered separately in early/non-cirrhotic MASLD and in advanced fibrosis or cirrhosis rather than presented as one uniform causal sequence.

#### 6.5.1 Early and non-cirrhotic MASLD: bidirectional metabolic reinforcement

In early or non-cirrhotic MASLD, visceral adiposity, insulin resistance, ectopic lipid accumulation, reduced physical activity, and muscle abnormalities share several upstream determinants and can reinforce one another. Prospective cohorts demonstrate that NAFLD/MASLD can precede incident sarcopenia or accelerated muscle loss (59, 60), whereas reduced muscle reserve may in turn decrease glucose-disposal capacity and worsen systemic and hepatic lipid flux (38, 41).

The most defensible interpretation is bidirectional metabolic reinforcement rather than a simple causal sequence. Muscle dysfunction is neither the sole driver nor a purely passive consequence; liver, adipose tissue, and muscle abnormalities evolve within a shared metabolic environment in which insulin resistance, ectopic fat, and inactivity reinforce one another. Residual confounding by obesity, T2D, diet, and activity still limits strong causal inference.

#### 6.5.2 Advanced fibrosis and cirrhosis: predominantly liver-driven muscle loss

In advanced fibrosis and cirrhosis, directionality increasingly shifts toward liver-driven muscle loss. Portosystemic shunting and impaired hepatic urea-cycle capacity increase skeletal muscle ammonia clearance, while glutamine synthesis, mitochondrial stress, proteolysis, and impaired protein synthesis contribute to anabolic resistance and wasting (41, 55, 88, 91, 92, 99). Evidence is strongest in cirrhosis; the clinical relevance of hyperammonemia in early non-cirrhotic MASLD remains uncertain.

Persistent injury and systemic inflammation may further increase muscle proteolysis, while reduced appetite, inadequate protein intake, fatigue, decompensation, and recurrent hospitalization reduce anabolic substrate and activity (39, 55, 88, 91, 92, 99). Muscle abnormalities in this setting increasingly reflect direct consequences of liver failure and its complications.

Disease stage should therefore be explicit whenever causal language is used. Early MASLD is best described as a synergistic metabolic system, whereas advanced or decompensated cirrhosis increasingly involves liver-to-muscle mechanisms. Pooling these stages can mix cause, mediator, consequence, and measurement artifact.

Figure 2 integrates these mechanisms into a stage- and compartment-dependent model: early bidirectional metabolic reinforcement links adipose tissue, liver, and skeletal muscle; physiological ApoJ contributes to liver-to-muscle metabolic signaling, while pathological intracellular or liver-to-kidney ApoJ signaling illustrates disease-specific remote-organ effects; and advanced cirrhosis increasingly drives muscle loss through hyperammonemia, inflammation, nutritional insufficiency, and reduced activity.

**Figure 2:**
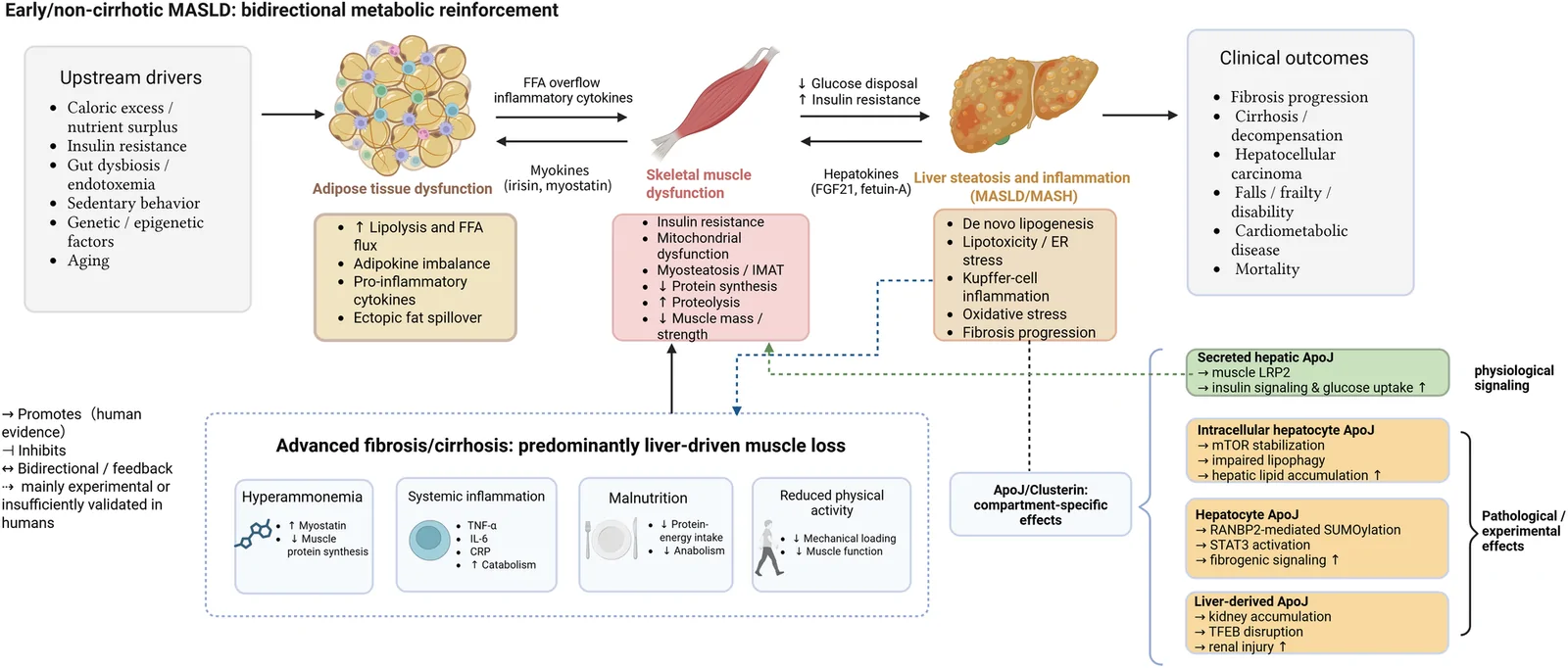
Stage-dependent liver–muscle–systemic crosstalk in metabolic dysfunction-associated steatotic liver disease (MASLD). In early or non-cirrhotic disease, visceral adiposity, hepatic lipid stress, muscle insulin resistance/myosteatosis, and reduced activity form a bidirectional metabolic network (38–43, 49, 59, 60, 70, 77, 96). Secreted hepatic apolipoprotein J (ApoJ) supports skeletal muscle insulin signaling through low-density lipoprotein receptor-related protein 2 (LRP2) (73), whereas intracellular hepatocyte ApoJ can stabilize mechanistic target of rapamycin (mTOR) and promote hepatic lipid deposition (74) or enhance RAN-binding protein 2 (RANBP2)–signal transducer and activator of transcription 3 (STAT3)-dependent fibrogenic signaling (75). Liver-to-kidney ApoJ accumulation can disrupt transcription factor EB (TFEB)-dependent lipid homeostasis and worsen diabetic renal injury (76). In advanced cirrhosis, hyperammonemia, inflammation, undernutrition, and inactivity increasingly make muscle loss a direct consequence of liver disease (64, 88, 91, 92). The figure does not imply that all ApoJ compartments are uniformly harmful. Created in BioRender. Ma, Z. (2026). https://BioRender.com/i61j62d

## 7 Assessment methods and minimum reporting standards for muscle and body-composition abnormalities

No single method captures muscle strength, physical performance, muscle quantity, myosteatosis, and adiposity simultaneously. Testing should therefore follow the construct and clinical question: functional tests assess strength/performance, BIA or DXA quantify muscle quantity and adiposity, and CT or MRI characterize anatomical muscle and fat. Results should be reported by the specific variable measured rather than under a nonspecific label such as “muscle quality.”

### 7.1 Assessment of muscle strength and physical performance

The Strength, Assistance with walking, Rise from a chair, Climb stairs, and Falls questionnaire (SARC-F) is a rapid sarcopenia screen but has limited sensitivity for early disease. Objective testing is therefore required when impairment is suspected. The revised European Working Group on Sarcopenia in Older People (EWGSOP2) uses low muscle strength to identify probable sarcopenia and impaired physical performance to indicate greater severity; the Asian Working Group for Sarcopenia (AWGS) 2019 likewise separates handgrip strength from performance measures such as gait speed, five-times sit-to-stand, and the Short Physical Performance Battery (SPPB) (22, 23).

Handgrip strength is inexpensive, reproducible, and associated with advanced fibrosis and mortality in MASLD (11, 25), but it predominantly reflects upper-limb strength and should not be assumed to represent whole-body function. This limitation is clinically relevant because upper- and lower-body criteria may identify different individuals. In the Tromsø study, the prevalence of probable sarcopenia was 1.3% when defined by handgrip strength and 4.4% when defined by chair-stand performance, with poor agreement between the two approaches (26). A normal handgrip result should therefore not automatically exclude clinically meaningful lower-limb or global functional impairment.

Assessment should follow the construct being evaluated. Handgrip is a practical strength measure, whereas five-times sit-to-stand, gait speed, or SPPB should be added when lower-limb or global physical performance is clinically relevant rather than described as interchangeable strength tests. This is particularly important with falls, cirrhosis, neuropathy, obesity, or mobility limitation.

Functional tests are influenced by pain, arthritis, neurological and cardiorespiratory disease, motivation, learning effects, and protocol variation. Reports should therefore specify the test, protocol, equipment, threshold source, and major comorbidities; strength and physical performance should remain distinct domains.

### 7.2 Assessment of muscle quantity and adiposity using BIA and DXA

Bioelectrical impedance analysis (BIA) is inexpensive, radiation free, and suitable for repeated outpatient assessment and has been widely used in NAFLD cohorts (15, 27, 100). Its principal limitation in liver disease is sensitivity to hydration status. Ascites, peripheral edema, recent food or fluid intake, and rapid changes in body water can bias estimates of lean and fat compartments. BIA results should therefore be interpreted cautiously in cirrhosis, hepatic decompensation, and periods of rapid weight change.

Dual-energy x-ray absorptiometry (DXA) provides relatively stable estimates of appendicular lean mass and total body fat percentage and is widely used in sarcopenia and sarcopenic obesity research (28, 30, 95). When combined with objective functional testing, DXA allows muscle quantity and function to be characterized separately. However, DXA does not directly quantify myosteatosis, and systematic differences across devices, software, and analysis algorithms can limit cross-platform comparison.

### 7.3 CT, MRI, and MRS assessment of muscle quantity and myosteatosis

Abdominal CT can characterize several body-composition compartments from a single examination. At the third lumbar vertebral (L3) level, CT can quantify skeletal muscle index (SMI), mean muscle attenuation, visceral adipose tissue, and subcutaneous adipose tissue, making it particularly useful for opportunistic analysis when abdominal imaging already exists (19, 29). However, CT attenuation is an indirect surrogate of muscle lipid rather than a direct biochemical measurement. Values are influenced by tube voltage, contrast phase, reconstruction method, segmentation technique, anatomical level, muscle selection, and threshold definition.

MRI provides more direct characterization of tissue fat without ionizing radiation. Dixon-based techniques can generate regional proton-density fat-fraction maps, whereas proton magnetic resonance spectroscopy (MRS) can quantify selected local lipid pools but is limited by small-volume sampling and more specialized acquisition and analysis requirements (20, 101–103). These modalities capture different biological levels and should not be treated as interchangeable measures of myosteatosis.

The accuracy of an indirect imaging modality should be considered at three distinct levels: analytic repeatability, agreement with an appropriate reference measure, and clinical validity for the intended outcome. These concepts should not be conflated. CT attenuation generally decreases as intramuscular lipid increases, but no universal Hounsfield-unit threshold is transferable across contrast phases, acquisition protocols, or populations (16, 20, 46). Automated segmentation can improve throughput and reproducibility, yet high algorithmic agreement does not eliminate calibration differences, spectrum bias, or uncertainty regarding the biological meaning of a threshold. Similarly, an association between CT attenuation and fibrosis or longitudinal liver-stiffness change establishes clinical relevance but does not demonstrate histological equivalence or prove that a change in attenuation represents improved muscle function (46, 104, 105).

MRI-based muscle fat quantification shows strong analytic performance under controlled conditions. Dixon MRI has shown correlations of approximately 0.95–0.97 with MRS for thigh-muscle fat (101), and mDIXON-Quant paraspinal measurements have shown intra- and interobserver intraclass correlation coefficients generally above 0.94 (102). Cross-center comparability nevertheless depends on field strength, sequence, anatomical region, region-of-interest definition, and software.

MRS is useful for localized lipid characterization but is limited by sampling volume and accessibility (101, 103). Muscle ultrasonography is portable and radiation free, but is operator- and probe-pressure dependent and lacks sufficient MASLD-specific prognostic validation.

No imaging index can substitute for objective strength or physical performance. Muscle quantity, muscle fat, strength, and performance should therefore remain separate variables in clinical reporting and research.

### 7.4 Opportunistic imaging, tool selection, and minimum reporting standards

Opportunistic body-composition analysis is relevant because many patients already undergo abdominal CT or MRI. Existing images can provide L3 SMI, muscle attenuation, visceral adipose tissue index and subcutaneous adipose tissue index without an additional scan. CT/MRI-derived muscle and body-composition measures have been associated with fibrosis in MASLD (16, 29), all-cause mortality (21), and selected outcomes in liver-transplant or hepatocellular-carcinoma cohorts (106–108). In liver-transplant cohorts, preoperative myosteatosis is also associated with complications, length of stay, and mortality (65–67).

A scalable workflow would combine automated segmentation, quality control, and clinical confirmation rather than universal manual analysis. However, imaging-selected patients often have more comorbidity or advanced disease than the wider MASLD population, and thresholds or automated models should be externally validated across scanners, protocols, institutions, and community populations before routine use.

Blood-based surrogates such as the creatinine-to-cystatin C ratio may supplement fibrosis assessment but cannot replace direct muscle measurements (109).

The major barrier to comparison remains standardization. Key sources of heterogeneity include CT/MRI acquisition and threshold definitions (16, 20), muscle-mass indexing (30), and differences in the constructs used for strength, sarcopenia, and “muscle quality” (47, 81–83, 110, 111). In particular, CT attenuation, MRI fat fraction, strength-to-mass ratios, and phase angle should not all be labelled “muscle quality.”

Studies should report the device and acquisition protocol, anatomical region, continuous values and categorical thresholds, threshold source, indexing method, population characteristics, and the specific muscle dimension measured. Whenever possible, strength, physical performance, muscle quantity, myosteatosis, visceral adiposity, and major confounders should be assessed concurrently.

Table 3 summarizes the principal assessment tools, their intended measurement domains, major limitations, and minimum reporting requirements.

**Table 3:** Tools for assessing body composition and muscle phenotypes: outputs, applicable settings, and minimum reporting requirements.

Tool	Core output	Advantages and applicable settings	Key limitations and minimum reporting requirements
SARC-F and objective functional tests	Grip strength, five-times sit-to-stand, gait speed, and SPPB.	Low cost and close to functional outcomes; suitable as the entry point for selective muscle assessment.	Affected by pain, motivation, and acute illness; report device, posture, number of attempts, summary method, and relevant comorbidities.
BIA	ALM/lean mass, fat mass, and phase angle.	Portable and radiation-free; suitable for repeat assessment in stable outpatients.	Affected by hydration, ascites, and recent food intake; report device, equation, measurement conditions, and indexing method.
DXA	ALM, regional lean mass, and body fat percentage.	Good repeatability; suitable for confirming the body-composition component of SO assessment and for longitudinal research.	Does not measure myosteatosis; report device, software, positioning, and indexing method.
CT (commonly at L3)	SMI, muscle attenuation, and visceral/subcutaneous adipose tissue.	Enables opportunistic analysis of existing abdominal imaging and concurrent assessment of muscle mass, muscle fat, and fat distribution.	Radiation and threshold heterogeneity; report anatomical level, kVp, contrast phase, reconstruction, segmentation, and threshold source.
Dixon MRI and proton MRS	Muscle fat fraction, regional distribution, and local lipid pools.	Radiation-free; suitable for repeated quantification and intervention trials.	Cost and sequence standardization limit use; report field strength, sequence, ROI/voxel, software, and repeatability.
Muscle ultrasonography	Muscle thickness, echo intensity, and pennation angle.	Bedside and radiation-free; suitable for people with mobility limitations.	Operator- and probe-pressure dependent; report probe, site, pressure, and repeatability; MASLD-specific validation remains insufficient.

ALM, appendicular lean mass; BIA, bioelectrical impedance analysis; CT, computed tomography; DXA, dual-energy x-ray absorptiometry; kVp, kilovolt peak; MRI, magnetic resonance imaging; MRS, magnetic resonance spectroscopy; ROI, region of interest; SMI, skeletal muscle index; SPPB, Short Physical Performance Battery. No single tool can replace concurrent assessment of function, muscle mass, and muscle fat.

## 8 Clinical integration of body composition With liver non-invasive testing and treatment monitoring

The clinical value of body-composition assessment in MASLD is to complement established liver-directed pathways with information on functional reserve, adiposity distribution, and treatment-related muscle vulnerability. Liver NITs estimate liver-disease severity, whereas strength, physical performance, muscle quantity, myosteatosis, and adiposity address distinct functional and metabolic domains that should remain clinically distinguishable.

### 8.1 Complementary value of body composition beyond liver non-invasive tests

Current MASLD pathways commonly use FIB-4 as a first-line assessment, with VCTE, ELF, or MRE used when fibrosis risk is indeterminate or elevated (5–7). These tools are central to liver-risk stratification but do not directly assess muscle reserve, physical performance, myosteatosis, frailty, or vulnerability during rapid weight loss.

Liver risk and muscle-related risk are therefore not equivalent. Associations of low strength, adverse muscle composition, and sarcopenia with fibrosis show overlap but do not make body-composition measures substitutes for liver NITs (16, 25, 112). A patient may have low current fibrosis risk but substantial functional or treatment-safety vulnerability, or conversely preserved muscle function despite a high-risk liver NIT.

Liver NITs also target different endpoints. The FibroScan–AST (FAST) score, which incorporates aspartate aminotransferase (AST), targets steatohepatitis with substantial activity and fibrosis (113), magnetic resonance elastography combined with FIB-4 (MEFIB) is used as a relatively specific rule-in strategy for fibrosis of at least stage 2 (114), and Agile 3 + and Agile 4 estimate advanced fibrosis and cirrhosis (115, 116). Serial interpretation may also be affected by biological and technical variability (117). None of these tools directly captures muscle-related vulnerability.

The key unresolved question is whether body composition adds clinically meaningful information beyond established liver pathways. Incremental value should therefore be tested against sequential FIB-4-to-VCTE/ELF/MRE strategies using calibration, discrimination, decision-curve analysis, and clinical net benefit rather than inferred from statistical significance alone.

Accordingly, liver and muscle-related risks should be reported separately rather than collapsed into an undefined “overall risk.” Table 4 summarizes their complementary roles.

**Table 4:** Complementary roles of liver non-invasive tests and body composition assessment.

Strategy/tool	Primary clinical question	Advantages	Key blind spots	Role in this review
FIB-4	Can advanced fibrosis be excluded at low cost?	Accessible in primary care; low values have high negative predictive value.	Affected by age and acute illness; broad indeterminate zone and limited positive predictive value.	Entry point for liver risk stratification; not a measure of muscle reserve.
FIB-4 → VCTE or ELF	How should the indeterminate zone be narrowed and referral/further evaluation decided?	Guideline-supported sequential pathway with relatively high clinical efficiency.	Still fibrosis-centered; availability is limited by equipment, cost, and technical failure.	Retained as the established core liver pathway.
MRE or composite NITs (FAST/MEFIB/Agile)	Specialist-level refinement, treatment eligibility, or liver-related prognosis.	Higher diagnostic performance in selected settings and may reduce unnecessary biopsy.	Targets and thresholds differ and are not interchangeable; function and muscle risk remain poorly captured.	For high-risk, discordant, or specialist settings.
Muscle function tests	Is low strength, activity limitation, or frailty present?	Low cost, close to functional outcomes, and repeatable.	Affected by pain, motivation, and comorbidity; does not assess fibrosis.	Selective screening in high-risk patients, not a liver NIT.
Body composition (BIA/DXA/CT/MRI)	Is low muscle mass, myosteatosis, or high visceral adiposity present?	Adds information on metabolic reserve, functional risk, and treatment safety.	Measurement and threshold heterogeneity; incremental value beyond liver NITs remains unestablished.	Third-tier selective assessment that does not replace current liver pathways.
Proposed integrated framework	How can liver stage, muscle phenotype, and adiposity status be reported jointly?	Covers liver risk, functional/nutritional risk, and vulnerability during weight loss.	Not externally validated; adds time, cost, and implementation complexity.	Hypothesis-generating framework requiring histological, longitudinal, and clinical-utility validation.

AST, aspartate aminotransferase; ELF, enhanced liver fibrosis; FAST, FibroScan–AST score; FIB-4, fibrosis-4 index; MEFIB, magnetic resonance elastography combined with FIB-4; MRE, magnetic resonance elastography; NIT, non-invasive test; VCTE, vibration-controlled transient elastography. Liver NIT pathways are based on references (5–7); representative evidence for FAST, MEFIB, and Agile is provided in references (113–116).

### 8.2 A selective four-step framework for clinical assessment

Routine comprehensive body-composition testing for every patient with MASLD is not supported by current evidence; a selective strategy is more appropriate.

Step 1: Establish the metabolic and adiposity context.

Initial assessment should characterize BMI, waist circumference, T2D, hypertension, dyslipidemia, age, recent weight change, physical activity, and other indicators of nutritional or functional vulnerability.

Step 2: Assess liver risk using established NIT pathways.

FIB-4 should remain the initial low-cost fibrosis assessment, followed when indicated by VCTE, ELF, MRE, or other validated liver-specific tests (5–7); body-composition assessment should not delay liver-risk escalation.

Step 3: Add selective muscle and body-composition assessment when clinically relevant.

Additional muscle assessment is most justified when it may alter risk characterization or management, including older age, T2D, normal weight with high metabolic risk, elevated liver NITs, cirrhosis, rapid weight loss, recurrent hospitalization, perioperative care, falls, or mobility limitation.

The minimum evaluation should include an objective strength or performance test plus an appropriate body-composition measure. Handgrip assesses strength, while five-times sit-to-stand, gait speed, or SPPB assess physical performance; BIA, DXA, CT, or MRI can characterize muscle quantity, adiposity, and—when appropriate—myosteatosis.

Step 4: Integrate liver stage, muscle phenotype, and adiposity status without conflating them.

Muscle findings should be classified according to A–D, whereas excess adiposity and liver stage are recorded independently. Phenotype C or D does not establish SO; “SO-related” should be used only when excess adiposity and the relevant SO criteria are independently confirmed.

A clinically useful summary could therefore be expressed as:

Liverstage×musclephenotype×adipositystatus.

This approach preserves the distinction between hepatic severity, muscle abnormality, and obesity-related risk rather than collapsing them into one composite label.

This four-step sequence is a conceptual framework rather than a guideline-endorsed algorithm. Its purpose should be specified, and histological, longitudinal, feasibility, cost-effectiveness, and clinical-utility validation are required before universal implementation.

Table 5 presents the proposed selective assessment pathway, while Figure 1 integrates the four-phenotype matrix with adiposity status and the clinical sequence.

**Table 5:** Proposed selective MASLD assessment framework enhanced by body composition.

Step	Core assessment	Entry condition or principal output	Potential management considerations
1. Baseline metabolic and adiposity assessment	BMI, waist circumference, T2D, blood pressure, lipids, age, physical activity, and recent weight change.	Identify obesity, metabolically high-risk normal weight, rapid weight loss, and other signs of muscle vulnerability.	Provide guideline-concordant lifestyle management and determine whether further liver or muscle assessment is needed.
2. Liver NIT stratification	Initial FIB-4; VCTE, ELF, or MRE for indeterminate or higher-risk results.	Generate low, intermediate, or high fibrosis risk and determine referral need.	Follow current liver-disease pathways for further testing, referral, treatment, and follow-up.
3. Selective muscle-phenotype assessment	At least one objective functional test plus one body composition tool; use existing CT/MRI opportunistically.	For older adults and people with T2D, normal-weight metabolic high risk, elevated NITs, cirrhosis, perioperative status, low activity, or rapid weight loss.	Identify functional, nutritional, and weight-loss-safety risks; consider nutrition, exercise, or rehabilitation referral and dynamic reassessment when abnormal.
4. Integrated phenotype reporting	Report the A–D muscle phenotype and separately record excess adiposity and liver stage.	Create a matrix of liver stage × muscle phenotype × adiposity status.	Use for research stratification and clinical communication; do not use alone to determine drug choice, liver biopsy, or fixed follow-up intervals.

BIA, bioelectrical impedance analysis; DXA, dual-energy x-ray absorptiometry; ELF, enhanced liver fibrosis; FIB-4, fibrosis-4 index; MRE, magnetic resonance elastography; NIT, non-invasive test; VCTE, vibration-controlled transient elastography. The framework has not been recommended by guidelines or prospectively validated for clinical utility and does not replace current liver-disease pathways.

### 8.3 Dual liver–muscle endpoints and treatment monitoring

Treatment monitoring in MASLD traditionally emphasizes liver outcomes such as alanine aminotransferase (ALT), controlled attenuation parameter (CAP), magnetic resonance imaging-proton density fat fraction (MRI-PDFF), liver stiffness, MASH resolution, and fibrosis improvement. These remain essential but may not capture muscle safety in patients with low reserve, SO, or myosteatosis.

A dual liver–muscle endpoint framework may therefore be useful when muscle safety is clinically relevant. Liver endpoints should match the intervention, while muscle endpoints remain construct specific: handgrip for strength; five-times sit-to-stand, gait speed, or SPPB for performance; appendicular lean mass or SMI for quantity; and CT attenuation or MRI muscle fat fraction for myosteatosis.

These measures are not interchangeable. A reduction in DXA-derived lean mass does not necessarily indicate deteriorating strength or performance, and improvement in liver fat does not guarantee preservation of muscle reserve.

Intervention studies only partly support this integrated approach. Lifestyle studies have assessed muscle quantity or objective muscle function alongside liver outcomes (118, 119). A separate energy-restriction and high-intensity interval-training trial assessed liver histology, body composition, cardiorespiratory fitness, and peripheral insulin sensitivity (120). Glucagon-like peptide-1 (GLP-1)-based studies increasingly report body composition, but standardized strength and performance endpoints remain uncommon (121, 122). Very few studies assess liver histology, strength, performance, muscle quantity, and myosteatosis concurrently.

Treatment effects should therefore not be judged solely from body-weight loss or absolute lean-mass change. Interpretation should consider baseline muscle reserve, the relative loss of fat and lean tissue, objective change in function, liver benefit, nutritional status, and the clinical context.

Monitoring intensity should reflect baseline vulnerability and treatment potency. Older patients, those with low baseline strength/performance, cirrhosis, rapid weight loss, or metabolic surgery may warrant structured reassessment using weight, waist circumference, one objective functional measure, and a body-composition measure. Fixed intervals should not be presented as validated MASLD-specific standards.

### 8.4 Treatment contexts requiring muscle-safety monitoring

Treatment is discussed here only to identify settings in which hepatic or metabolic benefit may coexist with clinically relevant muscle change. Table 6 summarizes the liver and muscle/body-composition outcomes of major interventions and their evidence boundaries.

**Table 6:** Liver and muscle/body composition outcomes of major interventions and their evidence boundaries.

Intervention	Representative evidence	Liver outcomes	Muscle/body composition outcomes	Evidence boundaries and practical considerations
Aerobic exercise	RCTs, meta-analyses, and consensus statements (123–128).	Improves liver fat, ALT/CAP, insulin resistance, and cardiorespiratory fitness.	Reduces visceral fat; preservation of muscle mass is inconsistent.	Appropriate for liver and cardiovascular risk reduction; patients with low baseline strength generally also need resistance training.
Resistance or combined exercise	RCTs, evidence syntheses, and intervention studies (118–120, 127–129).	May improve liver fat and metabolic indices, although effects are heterogeneous.	Improves muscle strength and selected lean-mass or functional outcomes.	Highly relevant to SO or rapid weight loss; no single optimal intensity or combination applies to all patients.
Caloric restriction or intermittent dietary regimens	RCTs, evidence reviews, and observational or secondary analyses (119, 120, 152–166).	Improves body weight, liver fat, CAP/MRI-PDFF, and selected histological outcomes.	Reduces fat mass but may also reduce fat-free mass.	No dietary pattern has shown consistent long-term superiority for muscle preservation; protein and resistance strategies should be individualized.
GLP-1 and multi-receptor agonists	Clinical trials, prospective studies, practice guidance, and systematic reviews (121, 122, 131–139).	Produces substantial weight loss and lower liver fat; some agents improve MASH-related outcomes.	Absolute lean mass may decline; standardized strength and physical-performance data are insufficient.	Do not equate lean-mass loss with sarcopenia; consider selective functional and body composition monitoring in older adults and those with low strength or rapid weight loss.
Resmetirom and FGF21 analogues	Drug trials (141–143).	Improves MASH, fibrosis, or liver-lipid-metabolism endpoints.	Direct endpoints for muscle mass, muscle fat, and muscle strength are scarce.	Muscle protection or harm cannot be inferred from a liver-targeted mechanism or hepatic benefit.
Metabolic surgery	Biopsy studies, cohorts, and reviews (144–146, 148).	Improves body weight, T2D, liver fat, and fibrosis-related outcomes.	Early postoperative lean-mass loss, nutritional deficiency, and fracture risk require attention.	Older adults and patients with cirrhosis, transplantation, or baseline SO merit preoperative screening and selective postoperative follow-up; uniform fixed monitoring is not supported.

ALT, alanine aminotransferase; CAP, controlled attenuation parameter; GLP-1, glucagon-like peptide-1; MRI-PDFF, magnetic resonance imaging-proton density fat fraction; RCT, randomized controlled trial; SO, sarcopenic obesity. The table provides practical interpretations within the boundaries of current evidence and does not constitute formal guideline recommendations.

#### 8.4.1 Lifestyle-Induced weight loss

Lifestyle-induced weight loss can improve liver fat, visceral adiposity, and insulin resistance, but muscle reserve matters when weight loss is rapid. Aerobic exercise reduces liver fat and improves cardiometabolic fitness (123–128), whereas resistance or combined exercise more directly improves strength and can help preserve lean tissue during caloric restriction (118, 119, 129). In patients with limited reserve, adequate nutrition and protein intake, resistance exercise, and objective functional monitoring should accompany weight reduction; cirrhosis additionally requires avoidance of prolonged fasting and adequate energy/protein intake (88, 91, 130).

#### 8.4.2 Pharmacological weight loss and liver-directed therapy

GLP-1 receptor agonists and related multi-receptor agonists can reduce body weight and liver fat (121, 131–133), and selected agents improve MASH-related outcomes (132, 134). Their body-composition effects require separate interpretation and have been synthesized in dedicated reviews (90, 122).

A meta-analysis in broader metabolic populations reported an average lean-mass reduction of 1.51 kg in GLP-1 receptor agonist trials; in pooled GLP-1 receptor agonist and sodium-glucose cotransporter-2 inhibitor analyses, lean mass accounted for 28% (95% confidence interval 22%–34%) of overall weight loss (135). However, a MASLD-specific systematic review did not show consistent clinically meaningful deterioration in muscle strength or composition (122); lean mass, muscle quantity, myosteatosis, strength, and performance are not synonymous endpoints.

A retatrutide DXA substudy showed reductions in both fat and lean mass without clear evidence of disproportionate lean-mass loss, but incomplete follow-up and absent standardized functional endpoints limit inference (136, 137). Tirzepatide, pemvidutide, and survodutide improve weight, liver fat, or MASH outcomes, while standardized muscle-function data remain limited (132, 133, 138, 139).

Preclinical work in obese mice suggests that exercise may partially preserve muscle mass and function during semaglutide treatment, but human confirmation is required (140).

Liver-directed therapies raise a different issue: resmetirom improves MASH and fibrosis-related outcomes (141), and FGF21 analogues such as pegozafermin and efruxifermin improve selected liver endpoints (142, 143), but standardized muscle outcomes remain scarce.

The ApoJ pathway provides an experimental therapeutic concept. Antagonizing pathological ApoJ chaperone activity promotes proteasomal mTOR degradation and reduces hepatic lipid deposition in preclinical models (74). Because physiological secreted ApoJ supports skeletal muscle insulin signaling (73), translation would require tissue- and isoform-selective targeting rather than nonspecific inhibition.

Future trials should therefore evaluate pharmacological efficacy together with muscle safety in clinically vulnerable subgroups.

#### 8.4.3 Metabolic surgery

Metabolic surgery can substantially improve obesity, T2D, and MASLD-related liver phenotypes, including histological outcomes (144, 145), while combined liver-transplant and sleeve-gastrectomy data provide selected evidence in transplant populations (146). Rapid changes in fat and lean compartments nevertheless make baseline muscle reserve and nutritional status relevant.

Preoperative SO is associated with adverse hepatic and cardiometabolic phenotypes and may improve after surgery, whereas persistent postoperative muscle abnormalities can accompany less favorable responses (144). High-risk patients may therefore benefit from preoperative functional and nutritional assessment.

Postoperative care should prioritize adequate protein intake, early mobilization, progressive resistance exercise, and selective body-composition follow-up. Prevention of malnutrition and sarcopenia is particularly important in cirrhosis (91, 130, 147), and bone health should also be considered because fracture risk varies by procedure (148).

Overall, body-composition assessment should be used selectively to identify functional vulnerability and preserve muscle safety while hepatic and metabolic disease is treated; its clinical utility still requires prospective validation.

## 9 A histology-anchored pathway for phenotype validation

The A–D framework is conceptual rather than validated. Prospective validation should preserve the distinction established in Section 4: A–D describe muscle status, excess adiposity is an independent modifier, and liver stage is a separate clinical dimension. The key question is whether this framework improves risk characterization beyond established liver NITs.

### 9.1 Study population and synchronous phenotype measurement

Initial validation should use a multicenter prospective cohort enriched for clinically indicated liver biopsy, with phenotype and adiposity measurements performed within a prespecified interval around biopsy.

Assessment should include objective muscle strength and/or physical performance, muscle quantity by DXA/BIA/CT, myosteatosis by CT attenuation or MRI muscle fat fraction, visceral adiposity, metabolic variables, and liver NITs including FIB-4, VCTE, ELF, or MRE.

Phenotypes A–D should be assigned from function/quantity and myosteatosis, while excess adiposity is recorded independently. This allows non-obese C/D phenotypes to remain distinguishable from SO.

Measurements should be performed without knowledge of histology whenever feasible, and synchronized closely enough to limit misclassification during rapid weight change, decompensation, or treatment.

### 9.2 Histological endpoints and pathological standards

Liver histology should serve as the research reference for MASH activity and fibrosis stage using a standardized scoring system, central blinded review by experienced liver pathologists, and prespecified adjudication of disagreement.

Primary cross-sectional endpoints may include histological MASH and clinically significant fibrosis; secondary endpoints may include steatosis, ballooning, inflammation, and fibrosis stage. Repeat biopsy should be limited to clinically or ethically appropriate settings.

Histology should test whether A–D correspond to different liver-disease patterns and whether independently defined excess adiposity modifies those associations, particularly for C/D phenotypes.

Biopsy is not a perfect reference because of sampling variability, interobserver disagreement, and selection bias. It should therefore anchor research validation but not be required solely for routine A–D assignment.

### 9.3 Model development and incremental value

Validation should not assume prognostic value. Initial analyses should compare histological outcomes across A–D and then test associations after prespecified adjustment for age, sex, T2D, visceral adiposity, physical activity, alcohol exposure, liver stage, and other relevant confounders.

Because excess adiposity is independent of A–D, models should examine muscle phenotype, adiposity status, and—when prespecified and adequately powered—their interaction. Interaction analyses should otherwise remain exploratory.

The principal clinical test is incremental value beyond a base model containing FIB-4 and relevant second-line NITs such as VCTE, ELF, or MRE.

Incremental value should be assessed with calibration, discrimination, decision-curve analysis, clinical net benefit, and internal validation rather than *P* values alone.

A significant phenotype coefficient is insufficient for clinical utility unless the framework improves prediction or decision-making beyond conventional metabolic variables and liver NITs.

### 9.4 From histological validation to longitudinal and clinical utility

Validation should proceed through four sequential stages.

Stage 1: Multicenter histology-anchored development.

A multicenter biopsy cohort should establish whether A–D correspond to distinct histological patterns and whether adiposity modifies these relationships using synchronized, blinded measurements.

Stage 2: Longitudinal validation of phenotype transitions and clinical outcomes.

A larger longitudinal cohort should test phenotype transitions against fibrosis progression/regression, decompensation, mortality, functional decline, and treatment-related muscle loss; repeat biopsy should be limited to appropriate settings.

Stage 3: External and multiethnic validation.

External validation should test transportability across countries, ethnic groups, sexes, disease stages, and levels of care, including non-obese MASLD and cirrhosis.

The conventional FIB-4-to-VCTE/ELF/MRE pathway should then be compared with the same pathway augmented by muscle phenotype and adiposity using calibration, discrimination, decision curves, resource use, and net benefit.

Stage 4: Pragmatic evaluation of clinical utility.

A pragmatic trial should be considered only after stable incremental value is demonstrated, comparing standard NIT-guided care with selective muscle/body-composition assessment used to guide functional, nutritional, or treatment-safety management.

The final question is whether using the framework changes clinically meaningful decisions or patient-important outcomes, not merely whether A–D is statistically associated with them.

Across all stages, biopsy should remain a research anchor rather than a prerequisite for phenotype assignment, and analyses should address selection bias, missing data, measurement error, and time-varying confounding.

Overall, validation should establish whether A–D correspond to different histological patterns, whether excess adiposity modifies these relationships, and whether integration with liver NITs improves prediction or decisions. Until then, A–D remains a research and communication framework rather than a validated diagnostic or prognostic algorithm.

## 10 Discussion: limitations and future research directions

This review provides an integrated framework for understanding the liver–muscle axis in MASLD by separating muscle phenotype, adiposity status, and liver-disease stage. The proposed A–D framework distinguishes muscle abnormalities based on function/quantity and myosteatosis while treating excess adiposity as an independent modifier, thereby avoiding misclassification of non-obese sarcopenia as sarcopenic obesity. The available evidence supports a bidirectional metabolic relationship in early MASLD and a progressively liver-driven pattern of muscle loss in advanced fibrosis and cirrhosis. However, several limitations remain before these concepts can be translated into validated clinical pathways.

First, this was a structured narrative review rather than a preregistered systematic review, and dual independent screening, formal risk-of-bias assessment, and quantitative pooling were not performed. The underlying literature is also highly heterogeneous in disease nomenclature, sarcopenia and SO definitions, muscle-mass indexing, imaging protocols, anatomical sites, thresholds, and outcome definitions (20, 30, 46, 64). These differences limit direct comparison among studies and complicate interpretation of whether one muscle dimension provides superior clinical information.

Second, causal inference remains challenging because most available studies are cross-sectional or rely on single baseline measurements. Visceral adiposity, physical activity, nutritional status, diabetes, and liver-disease severity may function as confounders, mediators, or both. Longitudinal studies nonetheless support time-dependent and potentially bidirectional relationships between liver disease and muscle/body-composition change (35, 59–63, 104). Furthermore, causal direction likely changes across disease stages: early MASLD is characterized by shared metabolic drivers and bidirectional reinforcement, whereas advanced cirrhosis involves additional liver-driven mechanisms such as hyperammonemia, inflammation, malnutrition, and reduced activity. Much of the evidence synthesized here comes from cohorts in East Asia, Europe, and North America (9, 10, 12, 33, 106, 149–151), so the transportability of thresholds across ethnicities, body sizes, and disease stages remains uncertain.

Third, current evidence does not yet validate the complete A–D framework as a prognostic classification system. Few cohorts simultaneously assess muscle strength, physical performance, muscle quantity, myosteatosis, visceral adiposity, liver histology, physical activity, and nutritional status. Therefore, available studies primarily support associations between individual muscle dimensions and clinical outcomes rather than demonstrating that specific phenotypes consistently predict different liver risks. Similarly, ApoJ provides important mechanistic insight into liver–muscle–systemic communication, but evidence regarding physiological secreted ApoJ, pathological intracellular ApoJ, and liver-to-kidney ApoJ signaling remains predominantly experimental (73–76).

Future research should prioritize standardized multidimensional assessment, prospective phenotype validation, and demonstration of clinical utility. A minimum core dataset should include objective muscle strength, physical performance when appropriate, standardized muscle quantity, myosteatosis, visceral adiposity, excess-adiposity status, physical activity, nutritional status, liver stage, and established liver NITs. The A–D framework should be evaluated in multicenter histology-anchored cohorts followed by longitudinal and multiethnic validation studies. Ultimately, the value of body-composition assessment should be determined not only by statistical association but by whether it improves clinical decision-making beyond existing liver NIT pathways through better calibration, discrimination, decision-curve performance, feasibility, and patient-important outcomes. Until such validation is achieved, body-composition assessment and the four-phenotype framework should be considered complementary tools for research and selective clinical characterization rather than replacements for established liver-risk stratification systems.

## 11 Conclusions

Sarcopenic obesity, non-obese sarcopenia or muscle insufficiency, and myosteatosis are related but non-equivalent manifestations of muscle and metabolic dysfunction in MASLD. Muscle strength, physical performance, muscle quantity, and muscle fat should therefore be evaluated as distinct constructs rather than collapsed into a single measure of “muscle quality.” The proposed A–D framework separates muscle function/quantity from myosteatosis and treats excess adiposity as an independent modifier. Accordingly, phenotypes C and D should be considered SO-related only when excess adiposity is independently confirmed and the relevant diagnostic criteria for SO are fulfilled; otherwise, they represent non-obese muscle insufficiency or sarcopenia, with or without coexisting myosteatosis.

Current evidence supports clinically relevant associations between adverse muscle status and MASH, fibrosis, mortality, and broader functional vulnerability, but no single muscle dimension has demonstrated consistent superiority across populations, disease stages, and outcomes. The liver–muscle relationship is also stage dependent: early or non-cirrhotic MASLD is best understood as a bidirectional metabolic system involving insulin resistance, ectopic lipid redistribution, inflammation, and mitochondrial dysfunction, whereas advanced fibrosis and cirrhosis increasingly introduce liver-driven mechanisms of muscle loss. ApoJ/clusterin further illustrates the compartment-specific nature of interorgan signaling, because physiological secreted hepatic ApoJ can support skeletal muscle insulin signaling, whereas pathological intracellular or remote-organ ApoJ accumulation may contribute to hepatic lipid deposition, fibrogenesis, and renal injury.

Body-composition assessment should therefore complement, rather than replace, established liver non-invasive testing. Its greatest potential value lies in identifying functional vulnerability, refining the interpretation of adiposity and muscle abnormalities, and monitoring muscle safety during potent weight-loss treatment. However, the A–D framework, selective assessment pathway, and dual liver-muscle monitoring strategy remain conceptual until they are validated against liver histology, longitudinal outcomes, diverse populations, and clinical utility. Future MASLD management should ultimately pursue three concurrent goals: protecting the liver, reducing harmful adiposity, and preserving muscle function.

## Glossary

ADAR2: adenosine deaminase acting on RNA 2
AdipoR1: adiponectin receptor 1
Akt: protein kinase B
ALM: appendicular lean mass
ALT: alanine aminotransferase
AMPK: AMP-activated protein kinase
ApoJ: apolipoprotein J/clusterin
AST: aspartate aminotransferase
AWGS: Asian Working Group for Sarcopenia
BIA: bioelectrical impedance analysis
BMI: body mass index
CAP: controlled attenuation parameter
CT: computed tomography
DXA: dual-energy x-ray absorptiometry
EASO: European Association for the Study of Obesity
ELF: enhanced liver fibrosis
ESPEN: European Society for Clinical Nutrition and Metabolism
EWGSOP2: revised European Working Group on Sarcopenia in Older People
FAST: FibroScan–AST score
FBW7: F-box and WD repeat domain-containing 7
FGF21: fibroblast growth factor 21
FIB-4: fibrosis-4 index
GABP: GA-binding protein
GLP-1: glucagon-like peptide-1
IL-6: interleukin-6
IMAT: intermuscular adipose tissue
LRP2: low-density lipoprotein receptor-related protein 2
MAFLD: metabolic dysfunction-associated fatty liver disease
MASLD: metabolic dysfunction-associated steatotic liver disease
MASH: metabolic dysfunction-associated steatohepatitis
MEFIB: magnetic resonance elastography combined with FIB-4
MetALD: metabolic dysfunction-associated alcohol-related liver disease
MRE: magnetic resonance elastography
MRI: magnetic resonance imaging
MRI-PDFF: magnetic resonance imaging-proton density fat fraction
MRS: magnetic resonance spectroscopy
mTOR: mechanistic target of rapamycin
NAFLD: nonalcoholic fatty liver disease
NASH: nonalcoholic steatohepatitis
NIT: non-invasive test
RANBP2: RAN-binding protein 2
ROR*α*: retinoic acid receptor-related orphan receptor alpha
SAA1: serum amyloid A1
SARC-F: Strength, Assistance with walking, Rise from a chair, Climb stairs, and Falls
SLD: steatotic liver disease
SMI: skeletal muscle index
SO: sarcopenic obesity
SPPB: Short Physical Performance Battery
STAT3: signal transducer and activator of transcription 3
T2D: type 2 diabetes
TFAM: mitochondrial transcription factor A
TFEB: transcription factor EB
TNF-α: tumor necrosis factor alpha
VCTE: vibration-controlled transient elastography.
